# Diagnostic laparoscopy for unexplained subfertility: a comprehensive review

**DOI:** 10.5935/1518-0557.20210084

**Published:** 2022

**Authors:** Wissam Arab

**Affiliations:** 1 Department of Obstetrics and Gynecology, Hôtel Dieu de France University Hospital, Saint Joseph University, Beirut, Lebanon

**Keywords:** laparoscopy, unexplained infertility, in vitro fertilization, endometriosis peritubal adhesions

## Abstract

Dealing with unexplained infertility is still non-guided, and patients are prone to different and sometimes discordant management strategies based on physician's preferences. However, much has been discussed in this matter, especially when it comes to the use of laparoscopy in patients with unexplained subfertility. In this debate article, we discussed data found in the literature concerning the utility of laparoscopy in these patients, leading us into establishing a new paradigm that will serve in orienting the physicians to when the procedure should be performed.

## INTRODUCTION

Treating infertile couples has largely evolved in the past few decades, with success rates of in vitro fertilization (IVF) increasing drastically in the last 40 years, while infertility assessment still undergoes updates and implementation of new tests of yet unproven effectiveness. The standard diagnostic evaluation of an infertile couple, after a 12-month period of regular unprotected intercourse without conception, aims at assessing the number of motile sperm in the male partner, certifying patency of at least one tube and documenting ovulation in the female partner (Practice Committee of the American Society for Reproductive Medicine, 2015). If these three conditions are satisfied, the infertility is said to be unexplained, meaning that there is no treatable cause so far identified. However, the technique for assessing each of these conditions remains till this day an area of disagreement, as well as the necessity of ruling out other conditions, for instance the existence of peritoneal factors (such as peritubal adhesions and endometriosis without tubal occlusion), before labelling the infertility as unexplained (Sadeghi, 2015). Many diagnostic tests used by clinicians managing unexplained subfertility remain of undefined role and relevance due to their high false positive rate, which means that their positivity might decrease but does not eliminate the possibility of a spontaneously occurring pregnancy. These tests include, among others, postcoital tests for cervical mucus evaluation, ovarian reserve assessment, endometrial biopsy, serum prolactine dosing, testing for immunological factors and performance of a diagnostic laparoscopy. In fact, unexplained infertility is not an absolute condition but rather a relative inability to conceive, and many of the couples may conceive without treatment (Isaksson & Tiitinen, 2004). On the other hand, the increased acceptance of assisted reproductive technology (ART) and its high success rates have made the route easy for clinicians as well as couples to relay on it, as an escape solution, in the absence of a treatable cause, avoiding a submersion into investigations and invasive treatments of unproven cost-effectiveness (Hatasaka, 2011). When approaching the assessment of tubal patency and peritoneal factors, most scientific committees concerned with infertility do not emit clear recommendations about the use of some investigative tools, especially laparoscopy. The Special Interest Group (SIG) in reproductive endocrinology is working under the ESHRE (European Society of Human Reproduction and Embryology) guidance on a project aiming to address the assessment and management of unexplained infertility. The guideline is expected in 2021.

The prevalence of unexplained infertility has reached 30% of infertile patients nowadays (Collins & Van Steirteghem, 2004). This rate might be lower if diagnostic tools with high detection rate would be implemented, detecting abnormal findings with proven impact on fertility. The role of laparoscopy and the timing of its use in this investigation remain an area of debate. Currently, the recommended empirical treatment of unexplained infertility includes three to six cycles of ovarian stimulation (OS) with oral agents (letrozole or clomiphene citrate) or gonadotropins associated with intrauterine insemination (IUI) ( Practice Committee of the American Society for Reproductive Medicine, 2020). OS-IUI improves fecundity by increasing the number of oocytes available for fertilization while increasing the number of motile sperm in the uterus. If this approach is unsuccessful, couples proceed onto IVF, preceded or not, by laparoscopy. A transformation is recently happening in the management of infertility after the advance of ART. The tendency is to move away from a diagnostic approach towards a prognostic approach, leading sometimes to the treatment of incompletely-evaluated patients (Balasch, 2000). Diagnostic laparoscopy is being increasingly bypassed before moving onto ART, in order to be cost-effective and to protect the women from the possible hazards of surgical and anesthetic complications (Bosteels *et al.*, 2007). The ease of access and the funding of ART by government programs and private health insurance plans in some countries has led to the acceleration of therapy towards ART in a straightforward fashion. IVF, when compared to OS-IUI, has been found to decrease the rate of multiple pregnancies using the elective single-embryo transfer technique (Bissonnette *et al.*, 2011). This finding has contributed as well to the acceleration of management towards IVF, obviating any conservative or diagnostic tentative, despite several critical analyses that tried to balance the arguments and save the place for a conservative approach in patients with unexplained infertility (Bahadur *et al.*, 2021).

Numerous challenges exist when analyzing the literature in relation to the effectiveness of laparoscopy in women with unexplained infertility. Published studies are numerous, but few are the randomized and controlled ones. The majority of the published evidence at present comes from prospective cohort studies. Few of these were able to correlate the effect of diagnostic findings and their treatment on the likelihood of conception. In addition, most studies did not include a control group, missing therefore the significant rate of unassisted pregnancies occurring with expectant management. Therefore, a systematic review on the utility of laparoscopy in unexplained infertility is not feasable. This led us to this debate article, trying to answer the following questions: how is the utility of laparoscopy defined in the context of unexplained infertility? By detecting more anomalies than hysterosalpingography (HSG), can we consider laparoscopy a useful test? Do these findings have an impact on the management plan and the fertility outcome, and if yes, in which patients?

## LAPAROSCOPY AND TUBOPERITONEAL DISEASE

Laparoscopy with direct visual examination of the pelvic anatomy is the ideal method available to diagnose tubal and peritoneal abnormalities that may impair fertility, in contrast with HSG which can miss pelvic adhesions and endometriotic implants (Fayez *et al.*, 1988; Hutchins, 1977; Rice *et al.*, 1986). When it was first implemented, laparoscopy was suggested as a mandatory step to rule out the existence of eendometriosis and peritubal adhesions as a cause of infertility, even when tubal patency with free spillage of injected dye has been demonstrated by HSG (Simon & Laufer, 1993). A great difference in the rate of abnormal findings was noted at that time between laparoscopy and the other noninvasive tests. In 1975, the first published paper concluded that laparoscopy frequently identifies a possible cause of infertility in women whose failure to conceive has remained unexplained by other methods of investigation (McDougall, 1976). A lesion might be identified in about 50 to 60% of cases of unexplained infertility (den Hartog *et al.*, 2008; Kanda *et al.*, 2006). The majority of these lesions are endometriotic ones, with accompanying tubal adhesions found in 20% of cases. Unilateral or bilateral tubal occlusion could still be found as well in a minority of cases (al-Badawi *et al.*, 1999; Bélisle *et al.*, 1996). However, many have questioned the real impact of such findings on fertility, by demonstrating the insignificance of pelvic adhesions and endometriotic implants in the presence of tubal patency: treating the lesions was associated with a small but insignificant increase in the likelihood of live birth (Thomas & Cooke, 1987).

In general, cumulative pregnancy rates of patients with unexplained pregnancy are high, weather treated with expectant management or with IUI/IVF. All management plans were proven to be equally effective when it comes to cumulative pregnancy rates, the median time to pregnancy being the only difference (Gunn & Bates, 2016). Nonetheless, the role of the peritoneal fluid and the spatial relationship between the tubes and the ovary in the fertilization process is well known. The volume of peritoneal fluid is significantly elevated in infertile women with endometriosis, as well as the levels of several cytokines such as IL-6 and TNF-a. Consequently, several adverse events occur, such as a reduction in sperm motility and a defect in granulosa cell steroidogenesis, contributing to endometriosis-associated infertility (Harada *et al.*, 1997; Harlow *et al.*, 1996; Yoshida *et al.*, 2004). These events have small but significant effects on birth rates that were demonstrated on larges samples. However, clinicians should not assume that they have solved the infertility if minimal or mild endometriosis was found and treated; these patients should remain classified as having an unexplained infertility. A Cochrane review in 2014 has demonstrated an improvement in ongoing pregnancy rates and live births after surgical treatment of minimal or mild endometriosis compared to expectant management. For each 24 women with early stage endometriosis treated by laparoscopy, an additional pregnancy can be obtained (Duffy *et al.*, 2014). Cumulative ongoing pregnancy rates could increase significantly, going from 18% in the control group to 31% in the treatment group (Donnez *et al.*, 2002; el-Yahia, 1994; Marcoux *et al.*, 1997). There is some evidence also that laparoscopic adhesiolysis with restoration of normal pelvic anatomy leads to an increase in cumulative pregnancy rates from 16% to 45% (Tulandi *et al.*, 1990). In women younger than 35 years, the cumulative pregnancy rate is higher after laparoscopic surgery compared to conservative management, including ART, but the mean duration of achieving pregnancy is longer (Nakagawa *et al.*, 2007).

Practitioners favoring IVF over conservative management with laparoscopy should not forget that endometriosis, even when minimal or mild, can adversely affect IVF outcomes (Barnhart *et al.*, 2002; Mahutte & Arici, 2001). Therefore, these conditions should be diagnosed and corrected before proceeding to IVF. In IVF failure patients, performing laparoscopy with fertility-enhancing surgical intervention can increase couple's fertility significantly, from 19.6 to 41.9 percent, with most of the patients conceiving spontaneously thereafter (Yu *et al.*, 2019). Completing the investigations with laparoscopy seems essential before undergoing IVF (Littman *et al.*, 2005). The main scientific committees remain reluctant to approve these findings so far. According to the ESHRE guidelines in 2005, minimal or mild endometriosis should be surgically treated when found, in order to improve fertility: the small impact that has been demonstrated on large groups of patients does not appear to justify a screening of all infertile women, considering the costs incurred and the risks associated with the surgical procedure (Kennedy *et al.*, 2005). There is a 1.84 risk of complication in every 1000 diagnostic laparoscopy performed (Chapron *et al.*, 2001). According to the American Society of Reproductive Medicine (ASRM), the impact of early stage endometriosis on fertility is relatively small, and most women with significant adnexal adhesions or advanced endometriosis have an abnormal HSG or, in the presence of normal HSG, have historical risk factors pointing towards a peritoneal factor (pelvic infection, surgery or pelvic pain). Still, the ASRM insist on the importance of considering peritoneal factors in women with otherwise unexplained infertility (Practice Committee of the American Society for Reproductive Medicine, 2015).

## LAPAROSCOPY AND TUBAL PATENCY

When managing a woman with infertility, it is important to avoid missing the correct diagnosis and treating a woman empirically for having an unexplained infertility, while existent tubal pathology might benefit of surgery. HSG has been considered as a screening test for tubal pathology, making only abnormal results an indication for laparoscopy to confirm the diagnosis, exclude artifacts resulting from transient tubal contractions and undergo a fertility-enhancing surgical intervention. However, many authors have stressed on the relative low sensitivity and the false negative rates when using HSG alone. Compared to laparoscopy, HSG has a sensitivity of 40 to 70% in the detection of bilateral tubal occlusion (Bélisle *et al.*, 1996; Broeze *et al.*, 2011). Contrast intravasation into uterine and ovarian veins can be mistaken for tubal filling, with a false negative rate reaching 50% in proximal tubal occlusion, and 60% in distal tubal occlusion (Ngowa *et al.*, 2015), the latter being accessible to surgical correction during laparoscopy. Proximal tubal occlusion can also be removed using a hysteroscopic cannulation under laparoscopic guidance (Honoré *et al.*, 1999). A meta-analysis of 20 studies in 1995 revealed that HSG has a sensitivity of 65% for the detection of tubal occlusion (Swart *et al.*, 1995). So, a normal hysterosalpingogram can give a false reassurance. We think that it would be prudent to consider HSG as a screening tool only in patients at low risk of tubal disease. When the prevalence of tubal pathology/occlusion becomes relatively high, a screening test with sub-optimal sensitivity might be confusing. Therefore, for those patients with a high risk of tubal disease, confirming tubal patency using a test with a higher sensitivity such as laparoscopy might be the most appropriate attitude.

New imaging techniques have been studied recently as diagnostic tools for assessing tubal patency with better results and single visits. Such techniques include air-saline hysterosalpingo-contrast sonography and hysterosalpingo-foam sonography with application of 3D imaging and the additional use of high definition flow (HDF) Doppler. In a prospective observational study, 2D-US using air or saline infusion showed a very high NPV (98-99.5%), making it useful as a screening test, while 3D-US with foam can be used to verify positive results due to its high diagnostic accuracy (Ludwin *et al.*, 2017). These techniques, despite their potential capacity to replace laparoscopy for the assessment of tubal patency, are not able to replace it in the evaluation of peritoneal factors (minimal or mild endometriosis) and peritubal adhesions (Swart *et al.*, 1995). Also, high level of evidence is still lacking and these techniques might not be reachable for all couples seeking infertility workup.

Not to forget that, similarly to HSG, these techniques are unable to screen for functional tubal abnormalities, in particular the presence of mild hydrosalpinges (Practice Committee of the American Society for Reproductive Medicine, 2006). Fallopian tubes can be patent under high pressures but dysfunctional physiologically (Karande *et al.*, 1995). Such abnormalities can be corrected during laparoscopy using neosalpingostomy, increasing therefore the pregnancy rates both spontaneously and with IVF (Yu *et al.*, 2018).

## ABNORMAL LAPAROSCOPY: WHAT ARE THE PREDICTIVE FACTORS?

In the above discussion, we demonstrated that using laparoscopy in a standard fashion for all patients with unexplained infertility will not add a major benefit to the outcome, taking into consideration the risks and expenses of the procedure (Buckett & Sierra, 2019). Therefore, predictive factors for an abnormal laparoscopy can be used as an indication for performing the procedure. The risk factors are those of tubal and peritoneal disease. Many factors have been proposed, including symptoms (such as dyspareunia or dysmenorrhea), chlamydia status and history of pelvic infection, secondary infertility, age and duration of infertility, history of previous surgery as well as the prior use of oral contraceptive pills (OCPs). Patient history is crucial in selecting patients requiring laparoscopy (Luttjeboer *et al.*, 2009; Portuondo *et al.*, 1984). Of women reporting dyspareunia, 51.7% had a positive laparoscopy (al-Badawi *et al.*, 1999). No prior use of OCPs was also predictive of abnormal findings, with a 64% likelihood ratio of pelvic adhesions (Capelo , 2003). Previous pelvic surgery increased the risk of abnormal findings: 72% of patients who had previously undergone various pelvic surgical procedures (gynaecological and non-gynaecological) had abnormal postoperative tubal sequelae (Musich & Behrman, 1982). A history of PID is associated with an increased rate of abnormal findings on laparoscopy (Opsahl & Klein, 1990). Chlamydia antibody titer (CAT) has as well a high discriminative capacity in the diagnosis of tubal pathology (Mol *et al*., 1997). The Dutch society of Obstetrics and Gynecology recommends the use of CAT as a first-line test in the basic work-up of subfertile couples, with a fixed cut-off level above which post-infectious pelvic disease should be ruled out using laparoscopy. Also, the rate of abnormal findings has been proven higher when comparing secondary to primary infertility (24 vs. 15%) (Hovav *et al.*, 1998). When considering these risk factors combined, 80% of women with negative CAT and a non-suspicious medical and surgical history will show no tubal pathology, so laparoscopy in these women can be deferred (Coppus *et al.*, 2007).

Duration of infertility was also correlated with the rate of abnormal findings on laparoscopy (Cundiff *et al.*, 1995). This was explained by the rate of spontaneous pregnancy that can occur meanwhile. In fact, some couples with unexplained subfertility are fertile but by chance did not conceive in the first year, or they have lesions with small impact on fertility; lesions that will be spontaneously overturned. When the female is less than 35 years of age, and the infertility period is short (1 year or less), the cumulative pregnancy rate can reach 80% in the first three years. After three years, the monthly conception rate decreases to 1-3%, with cumulative rates reaching 30% at best in couples with more than 5 years of infertility (Hull *et al.*, 1985). In this context, the ASRM advises to consider laparoscopy for young women with long duration of infertility (more than 3 years) (Practice Committee of the American Society for Reproductive Medicine, 2015). It has been demonstrated that in patients without risk factors and where infertility is of less than 3 years duration, expectant management is equally effective to OS/IUI and IVF, without a delay in time to conception (Bhattacharya *et al.*, 2001; Steures *et al.*, 2006). So, in patients with normal HSG and without any risk factor, laparoscopy should be delayed until expectant management fails. In this specific situation, the probability of finding clinically relevant abnormalities by laparoscopy will be higher; women of young age and without abnormalities should have become pregnant before laparoscopy. On the other hand, for patients with no identifiable predictor, delaying and even bypassing the procedure may be warranted (Fatum *et al.*, 2002). This attitude can reduce the costs of fertility treatment without compromising success rates. In its clinical guideline on fertility assessment and treatment, the National Institute of Clinical Excellence (NICE) in the United Kingdom advised the use of medical history to decide whether diagnostic laparoscopy should be performed or not (NICE, 2013). Some authors proposed using a multivariable prediction model including history and CAT, when deciding whether or not to perform a laparoscopy, in association with the woman's as well as the doctor's preferences (Coppus *et al.*, 2007). Although clinical history does not have the ultimate discriminative power to distinguish between patients with and without tubal/peritoneal pathology, we showed in this review that it can be used to create individualized patient risk profiles, especially when several factors are combined together.

Patients aged 35 or 38 might be an indication for bypassing laparoscopy and sometimes moving expeditiously towards IVF. There is good evidence that immediate IVF in women ≥ 38 years of age may be associated with a higher cumulative pregnancy rate as compared to a strategy consisting on expectant management or OS-IUI prior to IVF (Verhoeve *et al.*, 2013).

## LAPAROSCOPY AND THE MANAGEMENT PLAN OF UNEXPLAINED INFERTILITY

Utility of a diagnostic laparoscopy in unexplained infertility is skeptical, if it consists only on surgical correction of endometriosis and periadnexal adhesions. However, one of the most important arguments in favor of laparoscopy is the changement in the management plan that can result. Some of the abnormal findings during diagnostic laparoscopy can be severe enough to affect the woman's fertility and consequently the physician's management plan by excluding ovarian stimulation, thereby decreasing the emotional stress and financial burden resulting from unnecessary treatments (Corson *et al.*, 2000). This situation is estimated to occur in 25% of patients who would have been treated with OS-IUI: around a quarter of the patients with normal hysterosalpingograms might have endometriosis stage 3 and 4 or tubal occlusion (Bonneau *et al.*, 2012; Tsuji *et al.*, 2009). On the other hand, and as mentioned above, mild abnormal findings such as endometriosis stage 1-2, might not change the management plan but can permit surgical correction of lesions with mild effect on fertility, continuing afterwards with expectant management.

So, changing the treatment plan can occur from IVF to expectant management when mild findings are surgically corrected, or to IVF directly, bypassing therefore ovarian stimulation, in case of severe lesions.

## TIMING OF THE LAPAROSCOPY PROCEDURE

The optimal timing of laparoscopy is also a question that has been tackled, even in high risk patients. When predictive factors are found, laparoscopy performed before ovarian stimulation will orient the management into either surgical treatment followed by expectant management/OS-IUI or referral to IVF. Also, because treatment with OS-IUI requires optimal conditions for the ovum pick-up and its transport mechanism, diagnostic laparoscopy may be of value before progressing to IUI treatment. Performing the laparoscopy after failure of the OS-IUI cycles to detect more abnormalities due to a concentration effect was rejected (Tanahatoe *et al.*, 2005).

Performing a laparoscopy can also increase the conception rate in the first year post-laparoscopy, probably secondary to the mechanical effect of flushing the tubes, but the long term conception rate is unchanged if the laparoscopy is negative (Trimbos-Kemper *et al.*, 1984). Therefore, changing the management plan and proceeding to IVF should not occur before this 1-year time interval.

Concerning the interval between HSG and laparoscopy, three to six months are recommended, giving time to the positive mechanical effect of the HSG (Cundiff *et al.*, 1995).

## CONCLUSION

Traditionally, when tubal patency has been established by HSG, laparoscopy was suggested as a mandatory step to preclude the existence of pelvic pathologies as the cause of infertility (Rowe *et al.*, 1993; Watrelot *et al.*, 2003). However, it is difficult to persuade a woman with a normal HSG to undergo such an invasive procedure with high physical burden and exorbitant costs (Balasch, 2000). There is no consensus till today, between societies and trained infertility specialists, weather diagnostic laparoscopy should be adopted or not as a mandatory step before reaching the diagnosis of unexplained infertility. Voices have been raised against the exploitation of the infertile couple with expensive and unnecessary tests and procedures of unproven prognostic utility (Jaffe & Jewelewicz, 1991), while empiric treatment of unexplained infertility have reached high success rates with low costs and complications. The ESHRE Capri Workshop in 2000 stated that laparoscopy should be reserved as a further diagnostic procedure (Crosignani & Rubin, 2000). In a cost-effectiveness analysis, a computer-generated decision analysis tree was used to compare expectant management, standard infertility treatment, and laparoscopy with and without infertility treatment. The study concluded that laparoscopy followed by expectant management is cost effective in the management of young couples with prolonged (more than 3 years) and otherwise unexplained infertility (Moayeri *et al.*, 2009). Laparoscopy reveals the underdiagnosed pelvic pathologies that can contibute to subfertility, and can have a positive therapeutic effect on spontaneous conception by allowing surgical correction of these findings. However, its cost-effectiveness has not been demonstrated when applied systematically, and its effect on patients treated with OS-IUI later on is yet to be proven (Kamath *et al.*, 2020).

**Figure 1:**
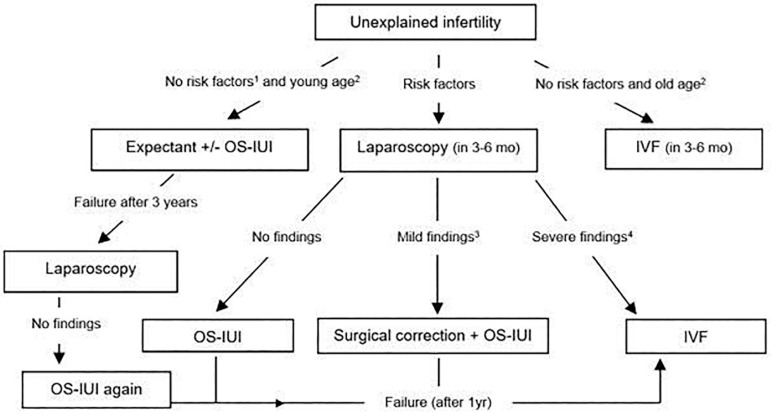
The algorithm proposed to illustrate the areas of utility of the procedure along the way of managing unexplained infertility. ^1^Risk factors include symptoms (dysmenorrhea, dyspareunia), previous pelvic surgery, secondary infertility, 3-year duration of infertility, previous PID or positive Chlamydia antibody titers, no OCP use. ^2^Young age is defined as age below 30 years, while old age above 35 years ^3^Mild findings included one-side thick adhesions or two-side filmy adhesions, minimal or mild endometriosis, one-sided phimosis, hydrosalpinx or tubal occlusion ^4^ Severe findings include moderate or severe endometriosis, two-sided hydrosalpinx/phimosis, bilateral dense adhesions and frozen pelvis.

We suggest that laparoscopy continues to be a useful tool in the workup of an infertile couple if performed on a case-by-case basis. Figure 1 shows the algorithm proposed by the author to illustrate the areas of utility of the procedure along the way of managing unexplained infertility. The algorithm was based on selection criteria, therefore avoiding the extensive use of the procedure, decreasing its costs and minimizing the diagnosis of minimal lesions of questionable prognostic significance.

An important issue to be raised as well in this context is the access and affordability of IVF treatments in low income countries, where patients face economic and legal barriers precluding IVF performance (Chiware *et al.*, 2021). In cases where IVF lacks availability, patients can be prone to undergoing an exploratory laparoscopy as an alternative route in order to look for eventual abnormal findings that could modify the fertility prognosis, even in cases with infertility of short duration and without predictive risk factors. A stepwise approach implementing the least expensive methods remain valid in couples with special circumstances. This additional factor was not mentioned in the algorithm, mainly for simplicity reasons.
